# Association between age at first calving, first lactation traits and lifetime productivity in Murrah buffaloes

**DOI:** 10.5713/ab.21.0182

**Published:** 2022-01-05

**Authors:** P. Tamboli, A. Bharadwaj, A. Chaurasiya, Y. C. Bangar, A. Jerome

**Affiliations:** 1 Indian Grassland and Fodder Research Institute, Jhansi, U.P. 284003, India; 2 Central Institute for Research on Buffalo, Hisar, Haryana 125001, India; 3 Department of Animal Nutrition, Nanaji Deshmukh Veterinary Science University, Jabalpur, M.P. 482001, India; 4 Animal Genetics and Breeding, Lala Lajpat Rai University of Veterinary and Animal Sciences, Hisar, Haryana 125004, India

**Keywords:** Association, Buffalo, Genetic, Heritability, Murrah, Phenotypic

## Abstract

**Objective:**

This study was conducted to estimate the association of age at first calving (AFC) with first lactation traits as well as lifetime performance traits in Murrah buffaloes.

**Methods:**

Data on first lactation and life time performance of Murrah buffaloes (n = 679), maintained at Indian Council of Agricultural Research-Central Institute for Research on Buffaloes, Hisar, India during the period 1983 through 2017, were deduced to calculate heritability estimates, genetic and phenotypic correlation of different first lactation and lifetime traits. The univariate animal model was fitted to estimate variance components and heritability separately for each trait, while bivariate animal models were set to estimate genetic and phenotypic correlations between traits under study.

**Results:**

The heritability was high for first peak milk yield (FPY, 0.64±0.08), moderate for AFC (0.48±0.07) and breeding efficiency (BE 0.39±0.09). High genetic correlations of first lactation total milk yield (FLTMY) with first lactation standard milk yield (FLSMY, 305 days or less), FPY, and first lactation length (FLL) was seen. Likewise, genetic correlation of AFC was positive with FLTMY, FLL, first dry period (FDP), first service period (FSP), first calving interval (FCI), herd life (HL) and productive days (PD). Significant phenotypic correlation of FLTMY was observed with HL, productive life (PL), PD, total lifetime milk yield (LTMY), standard lifetime milk yield (standard LTMY). Moreover, positive genetic and phenotypic correlation of FPY was observed with HL, PL, PD, total LTMY and standard LTMY.

**Conclusion:**

This study reports that AFC had positive genetic correlation with FDP, FSP, FCI, and unproductive days while, negative association of AFC was observed with FLSMY, PL, total LTMY, standard LTMY, and BE. This suggests that reduction of AFC would results in improvement of lifetime performance traits.

## INTRODUCTION

India possesses the largest buffalo population (109.85 million), which constitutes approximately 56.7% of total world’s buffalo population [1]. In addition, buffalo is the prime dairy animal contributing significantly to income, nutrition and employment for large rural population and hence considered as quadruple purpose animal i.e., suitable for milk, meat, draught and leather [2]. Sustainable viability of dairy enterprise depends on both production and reproduction potential of any livestock species, including buffaloes; however, livestock stake-holders neglect the fertility traits when compared to production traits [3,4]. In tropics, it has been well documented that buffaloes can efficiently utilize the roughages and crop by-products to produce high quality milk with high fat percentage and are resistant to many diseases [5]. Genetic make-up of animals governs performance of dairy farm and the knowledge of genetic and phenotypic parameters is of prime importance for designing suitable breeding policies to establish a genetically improved herd [6,7].

Selection on the basis of lifetime performance is not practically feasible due to long generation interval, so it is desirable to select the animals based on the performance of earlier lactations, rather than traits expressed later in life [8]. Likewise, suitability of breeding animals, including buffaloes, in an organized herd is primarily determined by its productive and reproductive efficiency [9]. First lactation traits are representative of subsequent lactation performance as these are highly inter-correlated both in cattle [10,11] and buffaloes [7,12]. Highly heritable traits can be used with great efficiency for genetic improvement through progeny testing as well as other genetic improvement programs. Thus, selection of animals based on the performance of the economically important traits having high heritability needs to be considered for overall improvement in lifetime productivity through correlated response [7,12].

Age at first calving is a prime reproductive trait affecting the herd’s productivity as well as profitability, through direct cost of rearing bovine heifers including buffaloes [13,11] and for its effect on future performance of the animal [10]. Shorter AFC prolongs the productive life (PL) in cattle [14] and buffalo [15]. Thus, dairyman would have a considerable interest in the relationship of AFC with lifetime production and other lifetime traits, as well. Furthermore, positive association between AFC and subsequent performance was observed suggesting the potential benefits of earlier calving in buffaloes [9,6]. Attempts to reduce the AFC helps in reducing production cost and increases the profitability of dairy enterprise [17]. Understanding the production and reproduction potential along with their inter-relationship is needed to formulate strategies in breeding programs in domestic animals, including buffalo [7,18,19]. Though attempts have been made to estimate the heritability, genetic and phenotypic correlations of the economic traits in buffalo [7,12, 20]. Further scope exist to analyse the inter-relationship of production and reproduction traits in buffaloes. Considering this aspect, the present study was designed to test the hypothesis of association between AFC, first lactation traits and lifetime productivity in Murrah buffaloes in organized herd.

## MATERIALS AND METHODS

### Collection of data

Data on first lactation and life time performance of 679 Murrah buffaloes at ICAR-Central Institute for Research on Buffaloes, Hisar that remained in the herd for at least three lactations were collected for the period from 1983 to 2017. The data was analysed to calculate genetic and phenotypic parameters and the data for all the studied animals were recorded up to the date of death or culling. Animals possessing records of minimum lactation length >150 days and total milk production >1,000 kg for the first lactation were considered for the present study. Further, animals with incomplete and abnormal records due to abortion, still birth, chronic illness etc. were excluded from analyses.

In this study, herd life (HL) was considered as duration from birth to disposal of animal and PL was defined as total days from date of first calving to date of start of last dry or date of disposal if animal in lactation. Likewise, the parameter productive days (PD) was denoted by the sum of the number of days in milk in different lactations in the same herd and unproductive days (UD) was defined by the sum of dry periods in different lactations in the same herd. In this study, lifetime milk yield was defined as total amount of milk produced by a buffalo from the initiation of first lactation till the last day in milk in the herd. It was considered for the animals that remained in the herd for at least three lactations. Milk yield per day of PL was measured as lifetime milk yield divided by the PL and milk yield per day of PD is the ratio of lifetime milk yield and PD. Likewise, milk yield per day of HL was the ratio between lifetime milk yield and HL.

In this investigation, the traits analysed were AFC in months, first lactation total milk yield (FLTMY) in kg, first lactation standard (305 days or less) milk yield (FLSMY) in kg, first peak milk yield (FPY) in kg/d, first lactation length (FLL) in days, first dry period (FDP) in days, first service period (FSP) in days and first calving interval (FCI) in days and lifetime performance traits such as HL in days, PL in days, PD in days, UD in days, breeding efficiency (BE) in %, total lifetime milk yield (total LTMY) in kg, standard lifetime milk yield (standard LTMY) in kg, milk yield per day of PL (MY/PL) in kg/d, milk yield per day of productive days (MY/PD)in kg/d, milk yield per day of herd life (MY/HL) in kg/d. The present research was approved by the Institute Animals Ethics Committee (IAEC).

### Statistical analyses

Data were analysed by least squares analysis model [21] using IBM SPSS Statistics 20.0 software to identify the significant fixed effects to be included in the model and determine associations between AFC, first lactation traits and lifetime productivity.

The least squares model for AFC included season of birth (4 levels) and period of birth (6 levels) as fixed effects. Fixed effects for first lactation traits were AFC (6 levels), season of first calving (4 levels), Period of first calving (6 levels); whereas for lifetime traits the fixed effects in least squares model were AFC (6 levels), season of first calving (4 levels), period of first calving (6 levels) and lactation completed (5 levels). The statistical significance was tested at 5% level and only the significant effects were included in the model that was subsequently used for analysis of genetic parameters.

The estimates of (co)variance components and genetic parameters were obtained via restricted maximum likelihood (REML) using Wombat software [22]. Convergence was assumed if difference in log likelihood function between consecutive iterations was lower than 5×10^−4^.

The univariate animal model was used in the study as follows:


y=Xβ+Zu+e

where *y* is the vector of records for different traits (FLTMY, total LTMY etc.); *X* is the incidence matrices assigning observations to fixed effects; *β* is the vector of fixed effects; *Z* is the incidence matrices assigning observations to random effects; *u* is the vector of random animal effects; *e* is the vector of random residual effects (error); further, bivariate analysis was performed between each pair of the first lactation and lifetime traits to estimate the genetic and phenotypic correlations.

## RESULTS

### Heritability estimates for first lactation and lifetime traits

The heritability for first lactation production and reproduction traits is presented in Table 1. The heritability was low for FLTMY (0.11±0.07), FLSMY (0.10±0.07), FLL (0.17±0.06), FDP (0.18±0.06), FSP (0.11±0.06), FCI (0.12±0.06). The estimate was moderate for AFC (0.48±0.07) and high for FPY (0.64±0.08). Likewise, heritability was low for HL (0.17±0.07), PL (0.08±0.07), PD (0.10±0.07), UD (0.06±0.07), total LTMY (0.17±0.07), standard LTMY (0.16±0.07), MY/PL (0.10±0.07), MY/PD (0.15±0.07), MY/HL (0.17±0.07), medium (0.2 to 0.4) for BE (0.39±0.09) (Table 2).

### Estimates of genetic correlation

#### Estimates of genetic correlation for first lactation performance traits

The genetic correlation for first lactation performance traits in Murrah breed is presented in Table 1 and 3. Genetic correlation of production traits (Table 1) were high and positive for FLTMY with FLSMY (0.603±0.276), FPY (0.661± 0.244) and FLL (0.653±0.307). Also, the genetic correlation of FLSMY was high and positive with FPY (0.908±0.253); while, medium and positive between FPY and FLL (0.307± 0.213).

Genetic correlation for reproduction trait were medium and positive for AFC with FSP (0.313±0.313) and FCI (0.308± 0.304); while, correlation of FDP was high and positive with FSP (0.694±0.222) and FCI (0.703±0.217); also, the correlation between FSP and FCI (0.972±0.041) was high and positive.

The genetic correlation between first lactation production and reproduction traits for Murrah buffaloes is presented in Table 3. Genetic correlation for FLTMY was positive with AFC (0.171±0.291), FSP (0.171±0.519); medium and positive with FCI (0.228±0.536); but negative with FDP (−0.283 ±0.369). Genetic correlation of FLSMY was medium and positive with FDP (0.308±0.654), but negative with AFC (−0.376±0.355). Moderate and positive correlation of FLSMY was found with FSP (0.448±0.692) and FCI (0.469±0.680). Genetic correlation of FPY was medium and positive with FSP (0.295±0.290) and FCI (0.365±0.289); moderate and negative with AFC (−0.423±0.139). The genetic correlation of FLL was medium and positive with AFC (0.297±0.232), FSP (0.215±0.296) and FCI (0.233±0.295) while moderate and negative with FDP (−0.528±0.325).

#### Estimates of genetic correlation among lifetime traits

Genetic correlation for HL was high and positive with PL, PD, UD, Total LTMY and Standard LTMY; moderate and positive with MY/PD; medium and positive with MY/PL, MY/HL and medium and negative with BE. Likewise, genetic correlation of PL was high and positive with PD, Total LTMY, Standard LTMY and MY/PD; while low and negative with BE. It is obvious that genetic correlation of PD was high and positive with Total LTMY (0.974±0.095) and Standard LTMY (0.939±0.111and low and positive with BE.

Genetic correlation of UD was moderate and positive with total LTMY, standard LTMY and MY/PD; whereas, moderate and negative with BE. Correlation of BE was low and positive with Total LTMY, standard LTMY, MY/PD, and MY/HL, medium and positive with MY/PL. The genetic correlation of total LTMY was high and positive with standard LTMY (0.986±0.040), MY/PD and MY/HL while, moderate and positive with MY/PL (0.585±0.379). High and positive correlation of Standard LTMY with MY/PL, MY/PD, and MY/HL was found. Association of MY/PL was medium and positive with MY/PD; but moderate and positive with MY/HL. Moderate and positive correlation observed between MY/PD and MY/HL (Tables 2, 4).

#### Estimates of genetic correlation between first lactation performance and lifetime traits

The genetic correlation of first lactation production and reproduction traits with lifetime traits of Murrah buffaloes is shown in Table 5 and 6. Genetic correlation of FLTMY was positive with HL, total LTMY, MY/PD while negative with UD. Likewise, genetic correlation of FLSMY was positive with HL, PL, PD, total LTMY, standard LTMY, MY/HL; but negative with BE and UD. Correlation of FPY was positive with HL, PL, total LTMY, standard LTMY, MY/HL, negative with BE, MY/PL, MY/PD.

Genetic correlation of AFC was positive with HL (0.058 ±0.192), negative with PL (−0.316±0.390), MY/PD (−0.281 ±0.280), PD (−0.494±0.352), BE (−0.426±0.187), total LTMY (−0.488±0.264), standard LTMY (−0.576±0.282), MY/PL (−0.441±0.371) and MY/HL (−0.496±0.237); moderate and positive with UD (0.403±0.492). Likewise, genetic correlation of FSP was medium and negative with PL, PD, total LTMY, standard LTMY, MY/HL; moderate and negative with BE, MY/PL. Furthermore, genetic correlation of FCI was medium and negative with PL, PD, BE, total LTMY, standard LTMY, MY/HL; moderate and negative with MY/PL.

### Estimates of phenotypic correlation

#### Estimates of phenotypic correlation for first lactation performance traits

Phenotypic correlation for first lactation production and reproduction performance traits in Murrah buffaloes is shown in Table 1 and 7. The phenotypic correlation of production traits in Murrah buffalo was high and positive for FLTMY with FLSMY, whereas medium and positive with FPY and moderate and positive with FLL (Table 1). The correlation of FLSMY was high and positive with FPY (0.526±0.031) and FLL (0.247±0.033) in Murrah buffalo. Phenotypic association of FDP was high and positive with FCI (0.743±0.019) and FSP (0.735±0.019) and also correlation between FSP and FCI (0.992±0.003) was observed to be high and positive.

In this study, the phenotypic correlation between production and reproduction traits of first lactation in Murrah is shown in Table 7. FLTMY was associated positively with AFC, FSP, FCI; but negatively with FDP. Phenotypic correlation of FLSMY was high and negative with FDP. Likewise, FPY was negatively correlated with AFC; and FLL was positively correlated with AFC, FSP, and FCI.

#### Estimates of phenotypic correlation between lifetime traits under study

Phenotypic correlation estimates among lifetime performance traits is presented in Table 2 and 8. Phenotypic correlation was high and positive for HL with PL, PD, UD, total LTMY, and standard LTMY; moderate and positive with MY/HL; medium and positive with MY/PL, MY/PD. Phenotypic correlation of PL was high and positive with PD, UD, total LTMY, and standard LTMY; moderate and positive with MY/HL; medium and positive with MY/PD. Correlation of PD was high and positive with UD, total LTMY, standard LTMY, and MY/HL; medium and positive with MY/PD. Phenotypic association of UD was highly positive with total LTMY and standard LTMY; medium and positive with MY/HL while, moderate and negative with BE, and BE was positively associated with total LTMY, standard LTMY, MY/HL, MY/PD, and MY/PL. Likewise, Phenotypic correlation among lifetime traits in Murrah buffaloes showed that total LTMY was high and positive with Standard LTMY and MY/HL; moderate and positive with MY/PD. Also, phenotypic correlation of standard LTMY was positively correlated with MY/HL, MY/PD, and MY/PL. Likewise, MY/PL was correlated with MY/HL and MY/PD.

#### Estimates of phenotypic correlation between first lactation performance and lifetime traits of Murrah buffaloes

Phenotypic correlation between first lactation performance and lifetime traits is shown in Table 9 and 10. Phenotypic of FLTMY was positively correlated with HL, PL, PD, total LTMY, standard LTMY, MY/PL, MY/PD, and MY/HL while negatively correlated with UD. Likewise, FLSMY was positively correlated with HL, PL, PD, total LTMY, standard LTMY, MY/PL, MY/PD, MY/HL, and BE. Moreover, FPY was high and positively correlated with HL, PD, total LTMY, standard LTMY, MY/PL, MY/PD, MY/HL, and PL. Furthermore, The FLL was positively correlated with HL, PL, PD, total LTMY, standard LTMY, MY/HL, MY/PL; whereas, negatively with UD, BE, and MY/PD.

Moreover, AFC was positively correlated with HL, MY/PL, and MY/PD and negatively with MY/HL. Likewise, FDP was highly and positively correlated with PL, UD, and negatively with MY/PL, MY/PD. In this investigation, it was observed that FSP was positively correlated with HL, PL, PD UD, total LTMY, standard LTMY and MY/HL; while, negatively with BE, MY/PL and MY/PD. Besides, FCI was positively correlated with PL, UD, and PD and negatively correlated with BE, MY/PL, MY/PD (Table 10).

## DISCUSSION

For dairy enterprise, production as well as reproduction parameters are pivotal for a profitability and sustainability. In this study, an attempt was made for estimating the association between AFC, first lactation traits and lifetime productivity in Murrah buffaloes in organized herd. Heritability estimates of the traits are useful for prediction of genetic response as well as the accuracy of selection [23]. In the present investigation, lower heritability estimates of production traits, FLTMY, FLSMY, FLL, FDP, FSP, FCI, total LTMY, LTMY, MY/PL, HL, MY/HL, and PL was in agreement with earlier reports [24–27] in buffaloes corroborating that these parameters, with low additive genetic variance, are less governed by genetic influences and henceforth, can be improved through better management. In buffaloes, lower to higher heritability for milk yield have been reported in different buffalo breeds [7,28–31] which suggests there is sufficient additive genetic variation to respond to selective breeding. These differences are probably caused by differences in locations and difference in genetic-makeup of the buffalo herd under study. Moreover, high heritability of trait such as FPY suggests that they can improve through a genetic improvement programme such as progeny testing.

The moderate heritability of reproductive traits i.e., AFC deduced in this study is supported by earlier workers [7,32, 33], in buffaloes, but lower heritability was observed in Murrah [34], Iranian [7], Mehsana buffaloes [23] along with Holstein Friesian cows [35]. The heritability for AFC indicates that this trait may respond reasonably well to selective breeding. A lower AFC is important because females that begin their reproductive life early can reduce production costs by increasing herd productivity. In one study [30], a lower estimate of 0.07 was obtained. In contrast, higher heritability estimates of AFC have been reported [36,37]. However, AFC is strongly influenced by the reproductive management of the herd and the lower heritability for reproductive traits except AFC in buffaloes indicate that much variation is attributed to the environmental factors and intervention through management is needed for improvement for this trait. These differences from the present investigation can be attributed to non-genetic factors environmental, breed, management, season, parity gradients used as well as the estimation methodology, which is a determinant factor in the reaction norm results [38].

With respect to genetic correlation among the first lactation performance traits, higher genetic correlation of FLTMY with FLSMY, FPY, FLL, and FLSMY with FPY and among other early performance traits hints that genes governing these traits might be common which needs to be studied in future [25]. Moreover, as FPY showed higher heritability, thus selection based on first peak yield might result in genetic improvement of other traits having high and positive genetic correlations with FPY, through correlated response [33]. The high genetic correlation between milk yield traits (FLSMY, FPY, and FLL) reported in this study implies that common genetic and physiological phenomenon affect the milk production trait. Also, a positive and high genetic correlation between the milk traits suggests the cumulative improvement of these traits, if used during selection [39].

Highly positive genetic correlation of FDP with FSP and FCI as well as FSP with FCI shows the increase in FDP and FCI with increase in FSP, since gestation period and lactation length tend to vary less in any species including buffaloes [25]. In contrast, lower genetic correlations of FPY with performance traits FSP and FCI have been reported in buffaloes [36,40]. Lower or negative genetic correlations between AFC with production traits i.e., FLTMY, FLSMY, FPY, and FLL are in agreement with earlier observations in buffaloes [7, 33,41]. As compared to this study, varied estimates of genetic correlation between AFC and LL, ranging from positive to negative have been reported by different workers in buffaloes [36,42]. Likewise, positive genetic correlation between AFC and CI is supported by [30]; but negative or absence of genetic correlations between AFC and CI in different breed of buffaloes have been observed [7,37]. These varied results can be attributed to different non-genetic parameters in addition to breed difference. In buffalo, the correlations (genetic and phenotypic) between MY and LL were moderate (from 0.47 to 0.52). Moderate to high genetic correlations (0.89 and 0.72) between these traits (production traits and LL) obtained in this study is constant with earlier reports [33,43]. These results corroborate that selection for milk yield traits could affect the lactation length confirming the commonness of genes influencing the traits which can be preferred for positive genetic gain. Likewise, higher genetic correlations between lifetime traits. HL, PL, PD, total LTMY, and standard LTMY as reported earlier in buffaloes [8,27] hints the selection of animals on the basis of these different lifetime traits can be advocated for genetic improvement in buffaloes. Genetic correlation between first lactation performance and lifetime traits indicates that these traits might be controlled by similar cellular pathways as well as genes [34, 44,45]. Due to positive genetic correlation for traits, selection for improvement in one trait will result in improvement in the other trait as a correlated response. Similar to our results, negative genetic correlation between AFC and PL in Murrah buffaloes was observed [46]. In accordance with our finding, positive genetic correlations of AFC with HL; FLTMY with HL, PL and total LTMY; FPY with HL, PL, and total LTMY was noticed [8,27]. AFC can be reduced through improved management practices i.e., nutritional management, health management, effective oestrus detection with insemination at the right time. Reduction in AFC to a definite level is desirable as it enhances the lifetime milk production traits total LTMY, HL, PL, and PD in buffaloes [9]. Furthermore, decrease in AFC decreases the cost of animal raising till the PL thereby increasing the annual genetic gain by decreasing the generation interval [10].

Regarding phenotypic correlation for first lactation performance traits, positive correlation between FPY and FLSMY shows the added weight given during selection as reported earlier in buffaloes [25]. Contrasting observation had been documented between production and reproduction traits in cattle [47] and buffalo [3,12,19] due to the genetic antagonism [48]. In cattle, several authors have reported improvement in milk production with improvement in reproduction in cattle [49–51], Brazilian Murrah [30,33,41].

In this study, positive phenotypic correlations between production traits (FLTMY, FDP, FPY, FLL) as well as reproductive trait (FCI, FSP) has been reported earlier in buffaloes [25,40,52] and cattle [53]. Likewise, it is noteworthy that in buffaloes, longer FSP was associated with longer FCI [40,52]. Contrarily in buffaloes, negative association of FLTMY with FLL and FDP [25] as well as negative correlation between AFC and FSP with FLTMY hinting the negative effect of milk production on reproduction [52].

About lifetime traits, positive phenotypic correlations of total LTMY with lifetime performance traits HL, PL, MY/PL, and MY/HL were in agreement with previous reports in buffaloes [8,27,54]. Similarly, positive phenotypic correlations of production (FLTMY) and reproduction performance (AFC) and lifetime traits (HL, PL, total LTMY, MY/HL, MY/PL, and UD) in buffaloes were in agreement with [8] and [27]. However, [55] reported that the herd life was not related to AFC in cattle breeds. On the contrary, negative phenotypic correlation (−0.08) between the two traits was reported in Murrah buffaloes [46] which was in agreement with our study. These variations in the outcome can be attributed to the management, environment diversity and genetic factors controlling these traits [56,57].

## CONCLUSION

In this study, heritability estimates were high, moderate and medium for traits FPY, AFC, and BE, respectively. Age at first calving had positive genetic correlation with FDP, FSP, FCI, HL, and UD while, it was negatively correlated with FLSMY, PL, total LTMY, standard LTMY and BE. A younger AFC is beneficial in that it can potentially lead to a reduction in rearing costs as well as an earlier return on investment. Hence our attempts should be made towards adoption of better management practices for proper growth and development of young heifers so that they conceive and first calve earlier. Genetic and phenotypic correlation of FPY was positive with FLTMY, FLSMY, FLL, HL, PL, PD, total LTMY, and standard LTMY. The selection of animals based on FPY should be considered because FPY had high heritability and is expressed earlier in the life, so choosing animals on the basis of peak yield may lead to improvement in other lifetime performance traits such as HL, PL, PD, total LTMY, and standard LTMY along with peak yield of the animal as a correlated response. The outcome of this investigation can be effectively utilized for future selection and breeding programs in buffalo species through considering high heritable and correlated traits of economic importance.

## Figures and Tables

**Table 1:** Heritability (diagonal), genetic (above diagonal) and phenotypic (below diagonal) correlation among first lactation traits in Murrah buffaloes

Traits	Production traits			

	FLTMY (kg)	FLSMY (kg)	FPY (kg/d)	FLL (d)
FLTMY (kg)	**0.11 (±0.07)**	0.603 (±0.276)	0.661 (±0.244)	0.653 (±0.307)
FLSMY (kg)	0.767 (±0.019)	**0.10 (±0.07)**	0.908 (±0.253)	0.134 (±0.332)
FPY (kg/d)	0.391 (±0.034)	0.526 (±0.031)	**0.64 (±0.08)**	0.307 (±0.213)
FLL (d)	0.554 (±0.026)	0.247 (±0.033)	0.017 (±0.037)	**0.17 (±0.06)**
	
	**Reproduction traits**			

	**AFC (mo)**	**FDP (d)**	**FSP (d)**	**FCI (d)**

AFC (mo)	**0.48 (±0.07)**	0.052 (±0.234)	0.313 (±0.313)	0.308 (±0.304)
FDP (d)	−0.089 (±0.035)	**0.18 (±0.06)**	0.694 (±0.222)	0.703 (±0.217)
FSP (d)	−0.033 (±0.036)	0.735 (±0.019)	**0.11 (±0.06)**	0.972 (±0.041)
FCI (d)	−0.038 (±0.036)	0.743 (±0.019)	0.992 (±0.003)	**0.12 (±0.06)**

FLTMY, first lactation total milk yield (kg); FLSMY, first lactation standard (305 days or less) milk yield (kg); FPY, first peak milk yield (kg/d); FLL, first lactation length (d); AFC, age at first calving (months); FDP, first dry period (d); FSP, first service period (d); FCI, first calving interval (d).

**Table 2:** Heritability (diagonal), genetic (above diagonal) and phenotypic (below diagonal) correlation among lifetime traits in Murrah buffaloes

Traits	HL (d)	PL (d)	PD (d)	UD (d)	BE (%)
HL (d)	**0.17 (±0.07)**	0.904 (±0.188)	0.823 (±0.171)	0.701 (±0.345)	−0.331 (±0.313)
PL (d)	0.863 (±0.014)	**0.08 (±0.07)**	0.967 (±0.098)	0.597 (±0.275)	−0.110 (±0.372)
PD (d)	0.873 (±0.014)	0.974 (±0.006)	**0.10 (±0.07)**	0.372 (±0.313)	0.011 (±0.858)
UD (d)	0.648 (±0.024)	0.840 (±0.016)	0.695 (±0.023)	**0.06 (±0.07)**	−0.435 (±0.412)
BE (%)	−0.015 (±0.042)	−0.177 (±0.041)	−0.062 (±0.042)	−0.411 (±0.037)	**0.39 (±0.09)**
	
	**Total LTMY (kg)**	**Standard LTMY (kg)**	**MY/PL (kg/d)**	**MY/PD (kg/d)**	**MY/HL (kg/d)**

Total LTMY (kg)	**0.17 (±0.07)**	0.986 (±0.040)	0.585 (±0.379)	0.646 (±0.286)	0.622 (±0.164)
Standard LTMY (kg)	0.989 (±0.004)	**0.16 (±0.07)**	0.615 (±0.410)	0.667 (±0.300)	0.658 (±0.160)
MY/PL (kg/d)	0.361 (±0.033)	0.334 (±0.033)	**0.10 (±0.07)**	0.380 (±0.243)	0.449 (±0.336)
MY/PD (kg/d)	0.450 (±0.030)	0.442 (±0.030)	0.517 (±0.023)	**0.15 (±0.07)**	0.424 (±0.273)
MY/HL (kg/d)	0.655 (±0.017)	0.659 (±0.017)	0.297 (±0.033)	0.334 (±0.031)	**0.17 (±0.07)**

HL, herd life (d); PL, productive life (d); PD, productive days (d); UD, unproductive days (d); BE, breeding efficiency; total LTMY, total lifetime milk yield (kg); standard LTMY, standard lifetime milk yield (kg); MY/PL, milk yield per day of productive life (kg/d); MY/PD, milk yield per day of productive days (kg/d); MY/HL, milk yield per day of herd life (kg/d).

**Table 3:** Genetic correlation between first lactation production and reproduction traits in Murrah buffaloes

Traits	AFC (mo)	FDP (d)	FSP (d)	FCI (d)
FLTMY (kg)	0.171 (±0.291)	−0.283 (±0.369)	0.171 (±0.519)	0.228 (±0.536)
FLSMY (kg)	−0.376 (±0.355)	0.308 (±0.654)	0.448 (±0.692)	0.469 (±0.680)
FPY (kg/d)	−0.423 (±0.139)	0.090 (±0.219)	0.295 (±0.290)	0.365 (±0.289)
FLL (d)	0.297 (±0.232)	−0.528 (±0.325)	0.215 (±0.296)	0.233 (±0.295)

AFC, age at first calving (months); FDP, first dry period (d); FSP, first service period (d); FCI, first calving interval (d); FLTMY, first lactation total milk yield (kg); FLSMY, first lactation standard (305 days or less) milk yield (kg); FPY, first peak milk yield (kg/d); FLL, first lactation length (d).

**Table 4:** Genetic correlation among lifetime traits

Traits	Total LTMY (kg)	Standard LTMY (kg)	MY/PL (kg/d)	MY/PD (kg/d)	MY/HL (kg/d)
HL (d)	0.788 (±0.154)	0.680 (±0.185)	0.325 (±0.425)	0.452 (±0.340)	0.313 (±0.270)
PL (d)	0.971 (±0.140)	0.924 (±0.143)	0.527 (±0.739)	0.619 (±0.515)	0.547 (±0.263)
PD (d)	0.974 (±0.095)	0.939 (±0.111)	0.574 (±0.566)	0.550 (±0.442)	0.588 (±0.225)
UD (d)	0.467 (±0.334)	0.405 (±0.318)	0.111 (±4.475)	0.522 (±0.645)	0.141 (±0.258)
BE (%)	0.039 (±0.286)	0.132 (±0.306)	0.320 (±0.296)	0.074 (±0.251)	0.169 (±0.295)

Total LTMY, total lifetime milk yield (kg); Standard LTMY, standard lifetime milk yield (kg); MY/PL, milk yield per day of productive life (kg/d); MY/PD, milk yield per day of productive days (kg/d); MY/HL, milk yield per day of herd life (kg/d); HL, herd life (d); PL, productive life (d); PD, productive days (d); UD, unproductive days (d); BE, breeding efficiency.

**Table 5:** Genetic correlation between first lactation production and lifetime traits in Murrah buffaloes

Traits	FLTMY (kg)	FLSMY (kg)	FPY (kg/d)	FLL (d)
HL (d)	0.535 (±0.477)	0.102 (±0.401)	0.135 (±0.221)	0.214 (±0.382)
PL (d)	0.258 (±0.642)	0.083 (±0.470)	0.118 (±0.317)	−0.067 (±0.881)
PD (d)	0.306 (±0.533)	0.242 (±0.548)	0.285 (±0.291)	−0.115 (±0.746)
UD (d)	−0.022 (±0.249)	−0.461 (±0.971)	−0.466 (±0.471)	0.116 (±1.934)
BE (%)	−0.304 (±0.392)	−0.153 (±0.423)	−0.105 (±0.167)	−0.332 (±0.256)
Total LTMY (kg)	0.131 (±0.326)	0.111 (±0.322)	0.153 (±0.209)	−0.210 (±0.436)
Standard LTMY (kg)	−0.013 (±177.369)	0.060 (±0.244)	0.103 (±0.212)	−0.314 (±0.445)
MY/PL (kg/d)	−0.088 (±11.977)	−0.048 (±110.595)	−0.021 (±2.142)	−0.314 (±0.618)
MY/PD (kg/d)	−0.216 (±1.717)	−0.207 (±2.621)	−0.190 (±0.305)	−0.184 (±0.396)
MY/HL (kg/d)	−0.163 (±1.403)	0.096 (±0.267)	0.045 (±0.173)	−0.446 (±0.453)

FLTMY, first lactation total milk yield (kg); FLSMY, first lactation standard (305 days or less) milk yield (kg); FPY, first peak milk yield (kg/d); FLL, first lactation length (d); HL, herd life (d); PL, productive life (d); PD, productive days (d); UD, unproductive days (d); Total LTMY, total lifetime milk yield (kg); Standard LTMY, standard lifetime milk yield (kg); MY/PL, milk yield per day of productive life (kg/d); MY/PD, milk yield per day of productive days (kg/d); MY/HL, milk yield per day of herd life (kg/d).

**Table 6:** Genetic correlation between first lactation reproduction and lifetime traits in Murrah buffaloes

Traits	AFC (mo)	FDP (d)	FSP (d)	FCI (d)
HL (d)	0.058 (±0.192)	−0.239 (±0.384)	−0.140 (±0.538)	−0.092 (±0.535)
PL (d)	−0.316 (±0.390)	−0.191 (±0.780)	−0.316 (±1.084)	−0.273 (±1.101)
PD (d)	−0.494 (±0.352)	−0.199 (±0.512)	−0.371 (±0.775)	−0.320 (±0.762)
UD (d)	0.403 (±0.492)	−0.070 (±8.736)	0.018 (±0.092)	0.014 (±0.072)
BE (%)	−0.426 (±0.187)	−0.167 (±0.217)	−0.404 (±0.254)	−0.468 (±0.247)
Total LTMY (kg)	−0.488 (±0.264)	−0.103 (±0.367)	−0.343 (±0.539)	−0.291 (±0.524)
Standard LTMY (kg)	−0.576 (±0.282)	−0.067 (±0.391)	−0.385 (±0.564)	−0.337 (±0.548)
MY/PL (kg/d)	−0.441 (±0.371)	−0.185 (±0.308)	−0.476 (±0.510)	−0.474 (±0.496)
MY/PD (kg/d)	−0.281 (±0.280)	−0.030 (±0.239)	−0.178 (±0.431)	−0.190 (±0.424)
MY/HL (kg/d)	−0.496 (±0.237)	0.096 (±0.434)	−0.259 (±0.533)	−0.263 (±0.520)

AFC, age at first calving (months); FDP, first dry period (d); FSP, first service period (d); FCI, first calving interval (d); HL, herd life (d); PL, productive life (d); PD, productive days (days); UD, unproductive days (d); BE, breeding efficiency; Total LTMY, total lifetime milk yield (kg); Standard LTMY, standard lifetime milk yield (kg); MY/PL, milk yield per day of productive life (kg/d); MY/PD, milk yield per day of productive days (kg/d); MY/HL, milk yield per day of herd life (kg/d).

**Table 7:** Phenotypic correlation between first lactation production and reproduction traits

Traits	FLTMY (kg)	FLSMY (kg)	FPY (kg/d)	FLL (d)
AFC (mo)	0.133 (±0.037)	0.026 (±0.037)	−0.076 (±0.040)	0.064 (±0.035)
FDP (d)	−0.310 (±0.033)	−0.218 (±0.034)	−0.058 (±0.037)	−0.259 (±0.032)
FSP (d)	0.104 (±0.036)	−0.029 (±0.035)	−0.053 (±0.038)	0.454 (±0.028)
FCI (d)	0.098 (±0.036)	−0.030 (±0.036)	−0.040 (±0.039)	0.454 (±0.028)

FLTMY, first lactation total milk yield (kg); FLSMY, first lactation standard (305 days or less) milk yield (kg); FPY, first peak milk yield (kg/d); FLL, first lactation length (d); AFC, age at first calving (mo); FDP, first dry period (d); FSP, first service period (d); FCI, first calving interval (d).

**Table 8:** Phenotypic correlation among lifetime traits in Murrah buffaloes

Traits	Total LTMY (kg)	Standard LTMY (kg)	MY/PL (kg/d)	MY/PD (kg/d)	MY/HL (kg/d)
HL (d)	0.863 (±0.015)	0.839 (±0.017)	0.229 (±0.036)	0.319 (±0.034)	0.434 (±0.030)
PL (d)	0.920 (±0.011)	0.924 (±0.010)	0.081 (±0.037)	0.224 (±0.035)	0.567 (±0.023)
PD (d)	0.952 (±0.008)	0.944 (±0.009)	0.185 (±0.036)	0.241 (±0.035)	0.600 (±0.021)
UD (d)	0.641 (±0.025)	0.670 (±0.024)	−0.186 (±0.036)	0.132 (±0.036)	0.363 (±0.032)
BE (%)	0.034 (±0.042)	0.071 (±0.042)	0.408 (±0.036)	0.240 (±0.040)	0.057 (±0.042)

Total LTMY, total lifetime milk yield (kg);Standard LTMY, standard lifetime milk yield (kg); MY/PL, milk yield per day of productive life (kg/d); MY/PD, milk yield per day of productive days (kg/d); MY/HL, milk yield per day of herd life (kg/d); HL, herd life (d); PL, productive life (d); PD, productive days (d); UD, unproductive days (d); BE, breeding efficiency.

**Table 9:** Phenotypic correlation between first lactation production and lifetime traits in Murrah buffaloes

Traits	FLTMY (kg)	FLSMY (kg)	FPY (kg/d)	FLL (d)
HL (d)	0.223 (±0.036)	0.152 (±0.037)	0.113 (±0.040)	0.051 (±0.035)
PL (d)	0.128 (±0.037)	0.122 (±0.037)	0.075 (±0.040)	0.046 (±0.035)
PD (d)	0.205 (±0.036)	0.174 (±0.036)	0.114 (±0.040)	0.113 (±0.035)
UD (d)	−0.084 (±0.037)	−0.028 (±0.036)	−0.036 (±0.040)	−0.125 (±0.034)
BE (%)	−0.032 (±0.041)	0.010 (±0.041)	0.053 (±0.044)	−0.287 (±0.037)
Total LTMY (kg)	0.300 (±0.035)	0.265 (±0.035)	0.169 (±0.039)	0.086 (±0.035)
Standard LTMY (kg)	0.248 (±0.036)	0.268 (±0.035)	0.167 (±0.039)	0.043 (±0.036)
MY/PL (kg/d)	0.454 (±0.030)	0.386 (±0.031)	0.173 (±0.038)	0.096 (±0.035)
MY/PD (kg/d)	0.371 (±0.033)	0.353 (±0.032)	0.174 (±0.038)	−0.023 (±0.035)
MY/HL (kg/d)	0.236 (±0.036)	0.245 (±0.035)	0.115 (±0.040)	0.065 (±0.036)

FLTMY, first lactation total milk yield (kg); FLSMY, first lactation standard (305 days or less) milk yield (kg); FPY, first peak milk yield (kg/d); FLL, first lactation length (d); FDP, first dry period (d); HL, herd life (d); PL, productive life (d); PD, productive days (d); UD, unproductive days (d); Total LTMY, total lifetime milk yield (kg); Standard LTMY, standard lifetime milk yield (kg); MY/PL, milk yield per day of productive life (kg/d); MY/PD, milk yield per day of productive days (kg/d); MY/HL, milk yield per day of herd life (kg/d); BE, breeding efficiency.

**Table 10:** Phenotypic correlation between first lactation reproduction and lifetime traits in Murrah buffaloes

Traits	AFC (mo)	FDP (d)	FSP (d)	FCI (d)
HL (d)	0.233 (±0.036)	−0.030 (±0.035)	0.004 (±0.036)	0.008 (±0.036)
PL (d)	−0.034 (±0.037)	0.125 (±0.035)	0.140 (±0.035)	0.147 (±0.035)
PD (d)	−0.046 (±0.037)	0.020 (±0.035)	0.091 (±0.036)	0.097 (±0.036)
UD (d)	0.000 (±0.038)	0.347 (±0.031)	0.225 (±0.034)	0.233 (±0.034)
BE (%)	0.023 (±0.041)	−0.406 (±0.034)	−0.550 (±0.031)	−0.574 (±0.031)
Total LTMY (kg)	−0.023 (±0.037)	−0.031 (±0.035)	0.028 (±0.036)	0.031 (±0.036)
Standard LTMY (kg)	−0.050 (±0.037)	−0.015 (±0.035)	0.014 (±0.036)	0.016 (±0.036)
MY/PL (kg/d)	0.075 (±0.037)	−0.338 (±0.031)	−0.225 (±0.034)	−0.245 (±0.034)
MY/PD (kg/d)	0.089 (±0.037)	−0.165 (±0.035)	−0.150 (±0.035)	−0.169 (±0.035)
MY/HL (kg/d)	−0.155 (±0.037)	−0.037 (±0.036)	0.015 (±0.036)	0.011 (±0.037)

AFC, age at first calving (mo); FDP, first dry period (d); FSP, first service period (d); FCI, first calving interval (d); HL, herd life (d); PL, productive life (d); PD, productive days (d); UD, unproductive days (d); BE, breeding efficiency; Total LTMY, total lifetime milk yield (kg); standard LTMY, standard lifetime milk yield (kg); MY/PL, milk yield per day of productive life (kg/d); MY/PD, milk yield per day of productive days (kg/d); MY/HL, milk yield per day of herd life (kg/d).
